# Deformation Resistance Performance of Carbon Fiber-Reinforced Plastic Machined by Controlling Drilling Area Temperature below the Glass Transition Temperature

**DOI:** 10.3390/ma14061394

**Published:** 2021-03-12

**Authors:** Chenping Zhang, Xiaohui Zhang, Yugang Duan, Yu Xia, Yueke Ming, Yansong Zhu

**Affiliations:** 1 School of Mechanical Engineering, Xi’an Jiaotong University, Xi’an 710049, China; zcp0824@stu.xjtu.edu.cn (C.Z.); ygduan@xjtu.edu.cn (Y.D.); mingyueke@foxmail.com (Y.M.); xjtu_zys@stu.xjtu.edu.cn (Y.Z.); 2 Research Institute of Aerospace Special Materials and Processing Technology, Beijing 100074, China; 13811058971@163.com

**Keywords:** carbon fiber-reinforced plastics, cryogenic machining, deformation resistance, glass transition temperature, drilling, damage analysis

## Abstract

Drilling of carbon fiber-reinforced plastics (CFRPs) is a challenging task in aviation and aerospace field. Damages, which can reduce the strength of the structure, often occur during secondary machining operations due to the applied cutting force and generated heat. The main objective of this study was to investigate the drilling performance and the deformation resistance of CFRPs subjected to cryogenic treatment based on glass transition temperature (Tg). Therefore, a cryogenic machining approach was adopted by fixing the workpiece inside a cryogenic box to drill CFRPs. The machining performance was briefly evaluated. Moreover, a through-hole drilling method was promoted to analyze the mechanism of different deformation mechanical properties. The results showed that the cryogenic machining approach improved the machining performance of CFRPs. Nevertheless, the residual intensity of cryo-treated specimen decreased (about 7.14%) due to the Tg-based viscoelasticity. These results demonstrate the great potential of this approach in advanced industrial applications and further pave the way for efficient secondary machining operation of CFRP components.

## 1. Introduction

Carbon fiber-reinforced plastic (CFRP), which consists of high-strength carbon fibers and matrices, is an excellent structural composite material. CFRPs have high stiffness and strength-to-weight ratio; thus, they have been widely used in many fields, such as aerospace, marine industries and civil engineering, automobile, robotics, wind-turbines, sport equipment, etc. [1,2,3,4,5] Nevertheless, despite their good mechanical properties, CFRPs exhibit some limitations. During the manufacturing of components from CFRPs, a secondary machining operation (turning, milling, drilling, etc.) is usually carried out after curing in order to meet the required tolerances and to manufacture fitting and joining surfaces [6,7,8]. During these machining operations of CFRPs, several damages such as delamination, fiber pull out, and matrix cracking may occur because of their non-homogenous and anisotropic properties and low thermal conductivity [9,10]. Moreover, the reliability of the assembling process may be reduced because of these damages. Furthermore, they can also lead to potential threats during service process of CFRP components. Thus, it is necessary to pay significant attention to the machining of CFRPs [11,12,13,14]. In addition to these damages mentioned above, heat resistance of the polymer matrix is also limited; thus, thermal damages and deterioration of the composite structure may occur due to the generation of heat during machining. However, mechanical properties and dimensional accuracy of the CFRP components may be affected by using conventional coolants [15,16,17,18,19,20].

In order to enhance the machinability of hard-to-cut materials such as soft aluminum, titanium, and abrasive composites, cryogenic machining has been adopted as an alternative approach to conventional machining. The high temperature generated during machining of CFRPs significantly affects the tool life, the machined surface quality, and the geometrical accuracy [1]. Therefore, the main objective of adopting cryogenic machining is to attempt to remove the effect of high temperatures generated during the machining process [21,22,23,24]. Moreover coolants can be extremely cold liquefied gases including oxygen, nitrogen, helium, or hydrogen in cryogenic machining. Among these coolants, liquid nitrogen (LN2) is the most used because of its environmental safety. In the literature, the majority of research employing the cryogenic machining method focused on turning titanium and steel alloys. Nevertheless, the investigation on cryogenic machining of CFRPs [25,26] has not been extensively reported and requires significant research attention.

Basmaci et al. [27] investigated the effect of cryogenic treatment and drill diameter on the drilling performance of CFRPs, for which the stacking sequence was 0°/90° orientation. CFRP laminate was fixed in a cryogenic bath filled with LN2 before and during the drilling process. Rajkumar et al. [28] carried out a comprehensive investigation on the delamination, thrust force, vibration, surface roughness, and fiber pull-out while drilling CFRP laminate under different feed rates and cutting velocities with dry and chilled air. Moreover, they proposed that the delamination factor could be reduced by the maximum of about 19.49% under chilled air environment. Samuel Raj et al. [29] monitored the wear of tools subjected to cryogenic treatment by measuring flank wear, peak flatting, cutting edge flatting, and cutting edge surface roughness. Kumar et al. [30] compared the machinability of conventional drilling of hybrid Ti/CFRP/Ti stack laminated in a single shot with or without cryogenic treatment. A significant improvement in hole quality under cryogenic condition was presented. However, the thrust force increased in cryogenic cutting because of the increased hardness of the Ti sheet under cryogenic treatment. Wang et al. [31] delivered the minimum quantity of lubrication coolants to the desired location on secondary cutting edges for effectively reducing tool wear of the secondary cutting edge corner. Ferreira Batista et al. [32] determined the effect of the cryogenic-treated and dry-treated drilling as well as tool feed rate of thermoplastic and thermoset CFRP on uncut fibers, delamination, roundness, and hole diameter. As a result, uncut fibers at the hole entry and delamination factor at the hole exit were reduced during cryogenic drilling for the thermoset CFRP [32].

However, the effect of cryogenic treatment on the mechanical properties of materials has been little studied. During the hole-making process of CFRPs, cryogenic CFRPs laminate cooling under cooled air was adopted in this study. Furthermore, the experiments were carried out with or without cryogenic treatment by using the same cutting parameters. Finally, the effect of cryogenic cooling on the deformation resistance properties was investigated using a fatigue-testing machine.

## 2. Materials and Methods

### 2.1. CFRP Laminates Manufacturing

**Figure 1:**
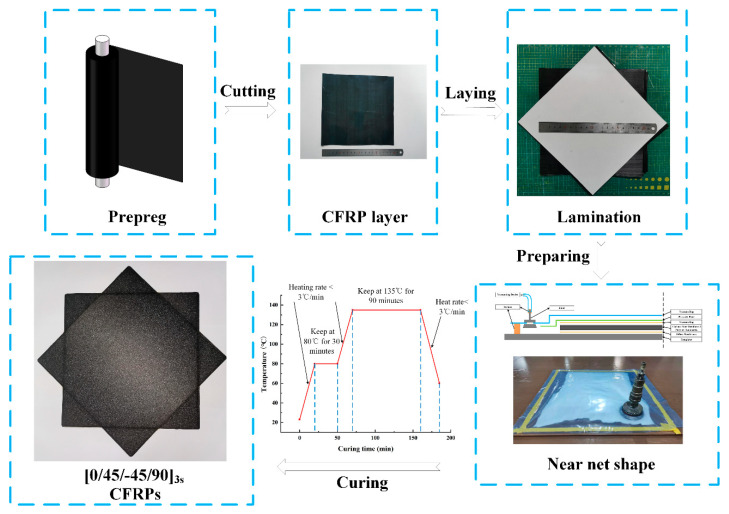
Schematic illustration of the manufacturing process of a carbon fiber-reinforced plastic (CFRP) plate.

CFRP laminates were manufactured using carbon T300/epoxy unidirectional prepregs, with ply thickness of 0.125 mm. The plate was manufactured by the vacuum-assisted resin transfer molding method. Moreover, the stacking sequence of the CFRP laminate was [45/0/−45/90]3s (totally 24 plies) with a total thickness of 3 mm. The manufacturing process of CFRP laminate is illustrated in Figure 1. The woven fabric preform was first cured at 80 °C for 30 min to complete the resin impregnation and then at 120 °C for 90 min. The dimensions of the laminate were 300 mm × 300 mm × 3 mm. Furthermore, the cured laminate was cut to meet the requirements of the American Society of Testing Materials (ASTM) standard.

### 2.2. Glass Transition Temperature of CFRP

The glass transition temperature (Tg), which defines the point at which the glassy polymers are transformed into flexible rubbers, determines the limited use temperature of CFRP laminates [33]. To verify the correlation between the CFRP’s deformation resistance property and drilling temperature, in this study, dynamic mechanical analysis (DMA) was used to obtain the Tg due to the mechanical properties of matrix being different at different temperatures. The DMA tests were conducted using a dynamic mechanical thermal analyzer (DMA242E, Netzsch, Germany) with three-point bending modes according to ASTM D7028. The dimensions of specimens are listed in Table 1.

In general, a lower heating rate yields more accurate results. In contrast, the drying property of CFRP laminates is affected by extremely low heating rates. Therefore, an appropriate heating rate of 5 °C min^−1^ was adopted to obtain accurate results. The heating process began at room temperature and stopped at 160 °C with an oscillation frequency of 1 Hz. To ensure the ratio of strain amplitude to maximum strain amplitude being in the range of linear viscoelasticity of CFRPs, the stress ratio was kept at 0.1%.

### 2.3. Experimental Design and Measurement

**Figure 2:**
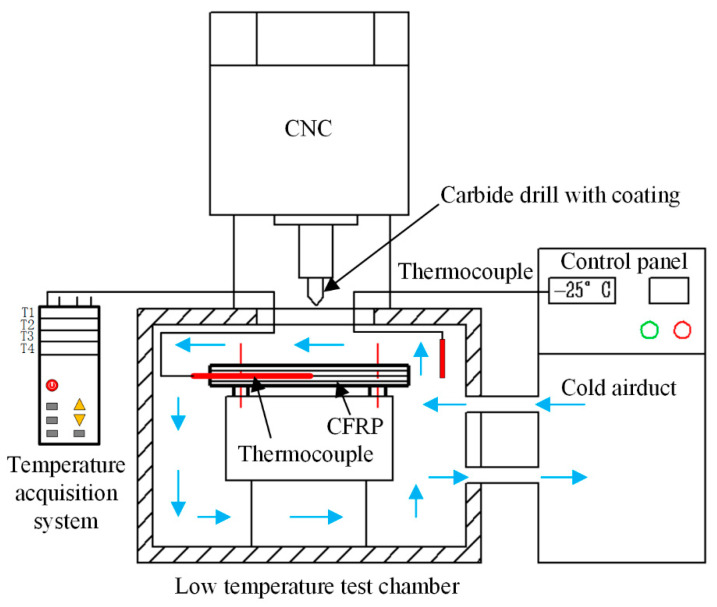
Schematic illustration of the temperature-controlled drilling experiment setup.

The backup support technique and the same cutting parameters (1000 rpm, 0.27 mm r^−1^) were used to obtain low and consistent damage specimens with uncoated carbide conventional twist drills (diameter 8 mm, point angle 118°) (Figure 2). To control the drilling area temperature (DAT), the cryogenic box was used and the relationship between environment temperature (ET) and DAT was investigated. The tensile/tensile and compressive/compressive fatigue tests were performed according to ASTM D5766 standard. The diameter of an open hole was 8 mm, and the width of the tested specimen was 48 mm maintain the ratio between width and diameter at six. The lengths of the specimen for tensile/tensile and compressive/compressive fatigue tests were 260 and 118 mm, respectively. The fatigue-testing machine (EHF-EV101k2-040-01A, Servo pulser, Kyoto, Japan) was used to obtain the results.

## 3. Results and Discussion

The glass transition temperature of CFRPs was obtained by the DMA test. Moreover, the relationship between ET and DAT was investigated to control the DAT during the hole-making process. Then, the drilled specimens were obtained by using consistent drilling parameters and backup support. Furthermore, tensile/tensile and compressive/compressive fatigue tests were performed. Finally, residual stress was measured to analyze the difference among the deformation resistance properties.

### 3.1. Glass Transition Temperature of CFRP

**Figure 3:**
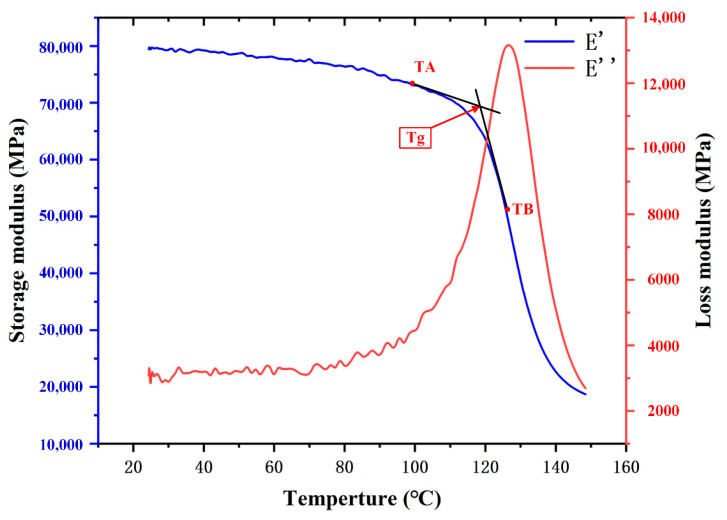
Representative curves of the loss modulus (right axis) and storage modulus (left axis) versus temperature for CFRP laminates measured by the dynamic mechanical analysis (DMA) test.

A large amount of heat was produced due to the friction between the tool cutting edge and an abrasive, which resulted in a decrease in the mechanical properties of the matrix. As a result, the damages occurred during the drilling process of CFRP [34]. Therefore, comprehensive understanding of the temperature-dependent mechanical properties of CFRP is essential. The result of the DMA is shown in Figure 3, where E′ represents the storage modulus and E″ represents the loss modulus. Moreover, TA is a beginning point of glass transition and TB is a mid-point of glass transition. Tg, the intersection of the tangent lines to points TA and TB, is 117 °C.

### 3.2. Drilling Test under Tg

**Figure 4:**
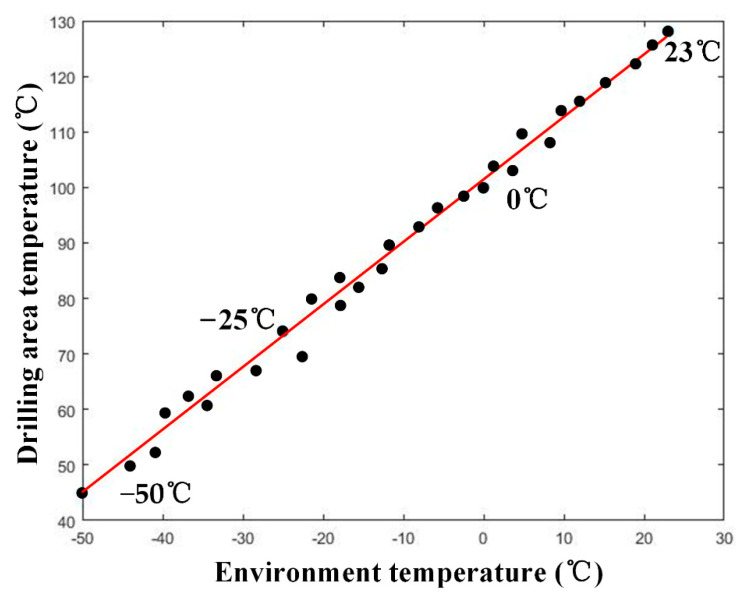
Mapping relationship between environment temperature (ET) and drilling area temperature (DAT).

During cryogenic machining applied in this study, the DAT was controlled by adjusting the ET in the cryogenic box. The effect of ET on DAT is shown in Figure 4. The result shows that the mapping relationship between ET and DAT is approximately linear, and *R*^2^ for the trend line is 99.1%. The linear relationship is represented in Equation (1):

(1) $$T_{D}=1.1266T_{E}+101.3957$$

where *T_D_* denotes the drilling area temperature (DAT) and *T_E_* represents the environment temperature (ET).

To investigate the mechanical properties of the CFPR specimen drilled at different temperatures, the cryogenic ET was −30 °C when DAT was 67 °C and lower than Tg; furthermore, a high ET was 40 °C, when the DAT was 146 °C and higher than Tg.

**Figure 5:**
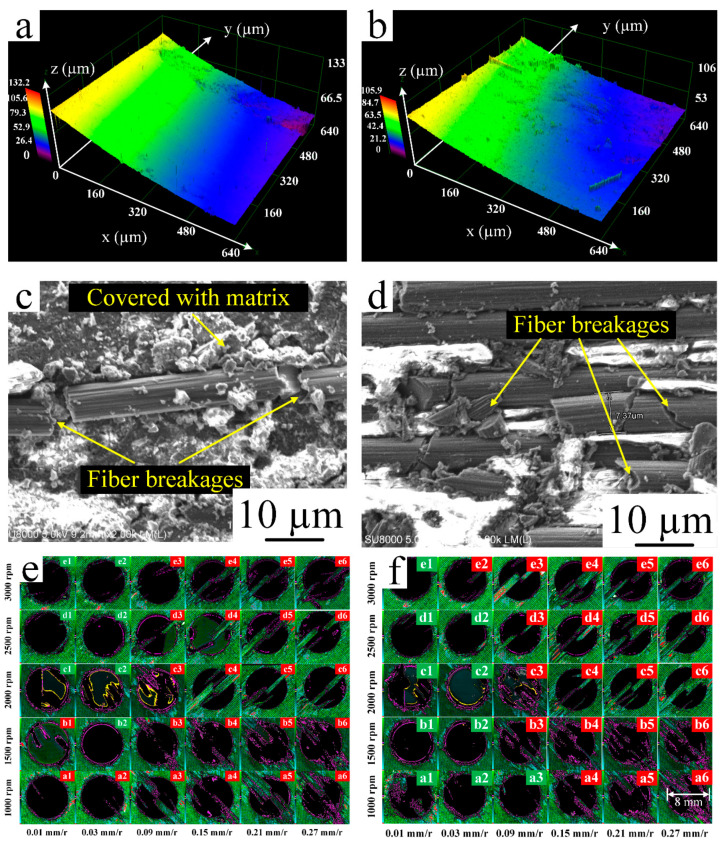
(**a**) Surface roughness of the hole drilled without cryogenic treatment; (**b**) surface roughness of the hole drilled with cryogenic treatment; (**c**) microstructure of the hole drilled without cryogenic treatment; (**d**) microstructure of the hole drilled with cryogenic treatment; (**e**) burr damage at the hole exit without cryogenic treatment; and (**f**) burr damage at the hole exit with cryogenic treatment.

The surface roughness, burr damages, and microstructure of holes drilled with or without cryogenic treatment are shown in Figure 5. The cryogenic treatment makes the specimens more brittle; moreover, the bonding force between molecules becomes stronger and the chemical bond between molecules shrinks [35]. Figure 5a,c demonstrate that Ra of the surface roughness decreases; conversely, the matrix covered increased compared to Figure 5b,d, which indicates an improvement in surface smoothness. Figure 5e,f show that the green box in the upper right corner of the hole represents less burr damage and that the red box in the upper right corner of the hole represents more burr damage; thus, burr damage decreases with cryogenic treatment.

### 3.3. Analysis of Deformation Resistance Performance

**Figure 6:**
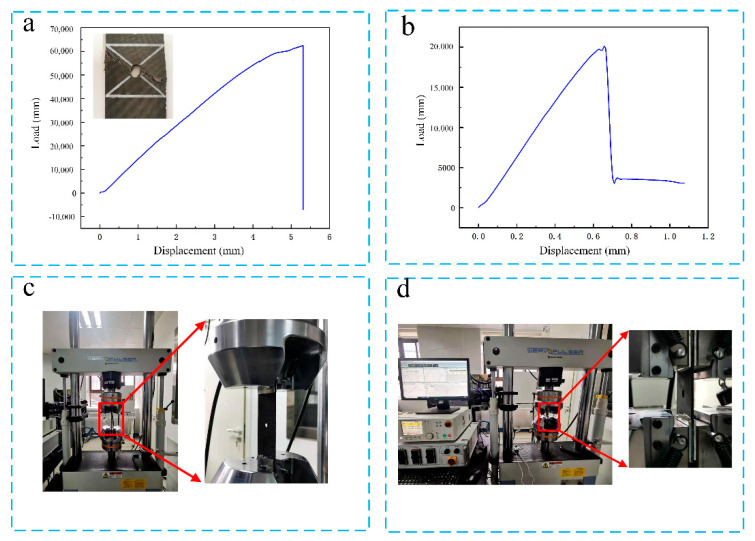
(**a**) Tensile test; (**b**) compressive test; (**c**) tensile/tensile fatigue experiment setup; and (**d**) compressive/compressive fatigue experiment setup.

To investigate the deformation resistance properties of a CFRP specimen drilled with or without cryogenic treatment, at least three specimens were tested according to ASTM D5766. The tensile/tensile fatigue test specimen dimensions were 260 mm × 48 mm × 3 mm. Moreover, the compressive/compressive fatigue test specimen dimensions were 118 mm × 48 mm × 3 mm. First, tensile test and compressive test were carried out (Figure 6a,b). Furthermore, load was applied during the fatigue test, which was the same for the untreated and treated specimens, and it was 70% of the ultimate load, where the applied loads during the tensile/tensile fatigue test and compressive/compressive fatigue test (*R* = 0.1 at *f* = 10 Hz) were 45.5 and 14 KN, respectively (Figure 6).

**Figure 7:**
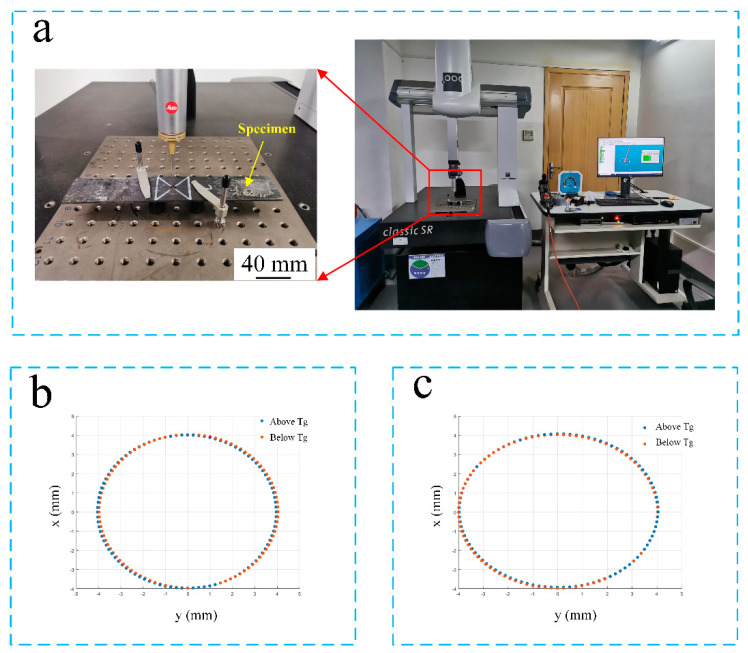
(**a**) Three-coordinate measuring machine used for testing deflection of a drilled hole after ten thousand cycles; (**b**) 2D distribution of a target point for tensile/tensile fatigue tests after ten thousand cycles; and (**c**) 2D distribution of a target point for compressive/compressive fatigue tests after ten thousand cycles.

Furthermore, a three-coordinate measuring machine (CS100 2828-18, classic SR. Germany) (Figure 7a) was used for measuring the deflection of a drilled hole after ten thousand cycles when the difference of deformation between untreated and treated specimens could be distinguished [36,37]. Notably, the roundness before fatigue tests remains the same. The results of deflection are presented in Figure 7b,c.

After applying ten thousand cycles of loading, the roundness of the tensile/tensile fatigue test specimen machined below Tg (−30 °C) decreased by 57% compared to the specimen machined above Tg (40 °C); reversely, the roundness of the compressive/compressive fatigue test specimen machined above Tg (40 °C) decreased by 76% compared to the specimen machined below Tg (−30 °C), as presented in Table 2. The results verified that the deformation resistance of a cryo-treated specimen is stronger than that of an untreated specimen after tensile/tensile loads are applied; nevertheless, weaker after compressive/compressive loads are applied.

Residual intensity is the ultimate tensile and compressive strength after ten thousand cycles. For testing the residual intensity of untreated and cryo-treated specimens after applying ten thousand cycles of loading, tensile tests and compressive tests were carried out, as presented in Table 3. After ten thousand cycles of tensile/tensile loads were applied, the residual intensity of specimens machined below Tg (−30 °C) slightly increased compared to those of specimens machined above Tg (40 °C); therefore, cryogenic treatment leads to a slight increase in the residual intensity. Nevertheless, residual intensity decreased by 7.14% after ten thousand cycles of compressive/compressive loads were applied.

### 3.4. Analysis of the Difference in Deformation Resistance Performance

**Figure 8:**
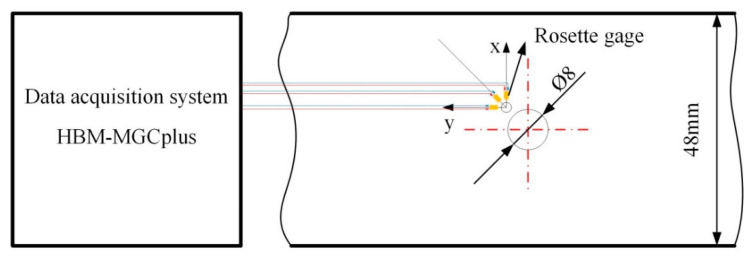
Schematic illustration of the through-hole drilling method.

Residual stress of untreated (above Tg) and cryo-treated (below Tg) specimens was tested to demonstrate that the divergence of deformation resistance property is caused by dissimilar viscoelasticity at temperatures below Tg or above Tg. For the residual stress analysis in orthotropic materials, the through-hole drilling method was applied in this study [38]. First, the CFRP plate was machined through untreated and cryo-treated methods. Second, a rosette gage (BF-120-2CA-K) was located onto the surface of the machined CFRP plate, with a distance of 1 mm from the drilled hole. Third, a through-hole, with diameter of 2 mm, was machined on the strain rosette (Figure 8). Finally, a data acquisition system (HBM-MGCplus, Darmstadt, Germany) received electrical signals from a rosette gage. Residual stress was obtained by calculating the strain acquired by breaking the balance inside the tested specimen.

**Figure 9:**
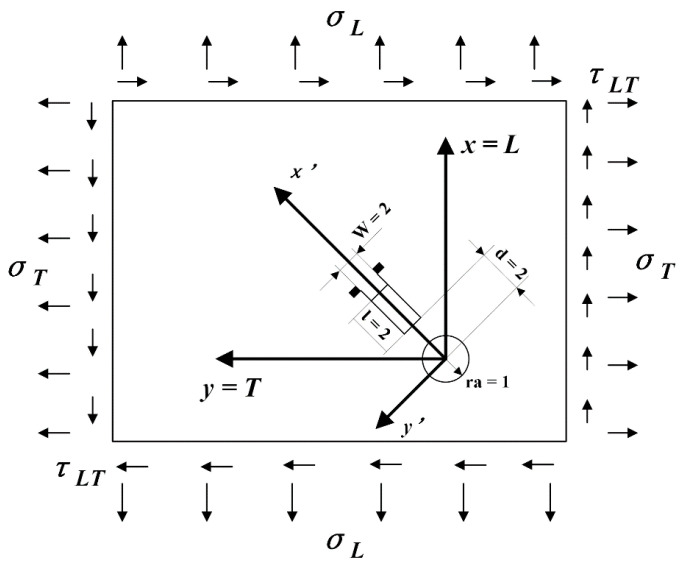
Schematic illustration of the plane coordinate system.

The mechanical properties of CFRPs with diverse layer directions are different because of the anisotropy feature. A plane coordinate system was established in this study (Figure 9), where direction *L* presents the 0° laying direction and direction *T* presents the 90° laying direction.

Therefore, the residual stresses of each layer are given by solving the following Equation (2):

(2) $$\left[\begin{array}{l} \sigma_{x}\\ \sigma_{y}\\ \tau_{xy} \end{array}\right]={\left[\tilde{E}\right]}_{k}{\left[\bar{E}\right]}^{-1}{\left[C\right]}^{-1}\left[\begin{array}{l} \varepsilon_{3}\\ \varepsilon_{2}\\ \varepsilon_{1} \end{array}\right]$$

In Equation (2), [*Ẽ*]*_k_* is the stiffness matrix of the *kth* ply (MPa), [*Ē*] is the dimensionless stiffness matrix, and [*C*] is the dimensionless influence coefficients matrix based on mathematical methods. Moreover, [*Ẽ*]*_k_* can be calculated by solving Equation (3):

(3) $${\left[\tilde{E}\right]}_{k}=\left[\begin{array}{ccc} \frac{E_{L}^{\left(k\right)}}{1-\nu_{LT}^{\left(k\right)}\nu_{TL}^{\left(k\right)}} & \frac{\nu_{LT}^{\left(k\right)}E_{L}^{\left(k\right)}}{1-\nu_{LT}^{\left(k\right)}\nu_{TL}^{\left(k\right)}} & \frac{\eta_{L,LT}^{\left(k\right)}}{G_{LT}^{\left(k\right)}}\\ \frac{\nu_{LT}^{\left(k\right)}E_{L}^{\left(k\right)}}{1-\nu_{LT}^{\left(k\right)}\nu_{TL}^{\left(k\right)}} & \frac{E_{T}^{\left(k\right)}}{1-\nu_{LT}^{\left(k\right)}\nu_{TL}^{\left(k\right)}} & \frac{\eta_{T,LT}^{\left(k\right)}}{G_{LT}^{\left(k\right)}}\\ \frac{\eta_{L,LT}^{\left(k\right)}}{G_{LT}^{\left(k\right)}} & \frac{\eta_{T,LT}^{\left(k\right)}}{G_{LT}^{\left(k\right)}} & G_{LT}^{\left(k\right)} \end{array}\right]\;(k=1,2\ldots n)$$

where $E_{L}^{\left(k\right)}$, $E_{T}^{\left(k\right)}$, $G_{LT}^{\left(k\right)}$, $v_{LT}^{\left(k\right)}$, and $v_{TL}^{\left(k\right)}$ are the elastic properties of the *kth* ply in the principal coordinate system *L−T* of the laminate; $E_{L}^{\left(k\right)}$ is the tensile modulus of the *kth* ply along the direction *L* (MPa); $E_{T}^{\left(k\right)}$ is the tensile modulus of the *kth* ply along the direction *T* (MPa); $G_{LT}^{\left(k\right)}$ is the shear modulus of plane *LT* (MPa); $v_{LT}^{\left(k\right)}$ is Poisson’s ratio along the direction *LT*; $v_{TL}^{\left(k\right)}$ is Poisson’s ratio along the direction *TL*, and ${ɳ}_{T,LT}^{\left(k\right)}$ is the dimensionless coefficient. 

It is necessary to determine the unknown strain vector {*ε*_3_, *ε*_1_, *ε*_2_} to evaluate the residual stress distribution in each ply according to Equation (2). Obviously, to this aim, the actual laminate can be advantageously replaced by the equivalent homogenous orthotropic material, which has the same in-plane elastic behavior as the actual laminate, for which the elastic properties are related to those of each ply by the following relationships [38]:

(4) $$E_{L}=\frac{\sum_{k}^{n}E_{L,k}s_{k}}{h}$$

(5) $$E_{T}=\frac{\sum_{k}^{n}E_{T,k}s_{k}}{h}$$

(6) $$G_{LT}=\frac{\sum_{k}^{n}G_{LT,k}s_{k}}{h}$$

(7) $$\nu_{LT}=\frac{\sum_{k}^{n}\nu_{LT,k}s_{k}}{h}$$

where *S_k_* is the thickness of the *kth* ply (mm), *h* is the total thickness of the CFRP plate (mm), and *n* is the number of layers. 

In Equation (2), [*Ē*], which is the dimensionless stiffness matrix, can be calculated by using Equation (8):

(8) $$\left[\bar{E}\right]=\left[\begin{array}{ccc} \frac{\bar{E}}{\bar{E}-{\bar{\nu }}^{2}} & \frac{\bar{\nu }}{\bar{E}-{\bar{\nu }}^{2}} & 0\\ \frac{\bar{\nu }}{\bar{E}-{\bar{\nu }}^{2}} & \frac{1}{\bar{E}-{\bar{\nu }}^{2}} & 0\\ 0 & 0 & \frac{\bar{G}}{\bar{E}} \end{array}\right]\;$$

where *Ē*, $\bar{G}$, and $\bar{\nu }$ are the dimensionless elastic constant coefficients of the *kth* ply on plane *LT*. Furthermore, $\bar{E}$, $\bar{G}$, and $\bar{\nu }$ can be calculated by using the following equations:

(9) $$\bar{E}=E_{L}/E_{T}$$

(10) $$\bar{G}=G_{LT}/E_{T}$$

(11) $$\bar{\nu }=\nu_{LT}$$

The mechanical properties of the CFRP plate are presented in Table 4.

The stiffness matrix of a typical lay-up direction with the principal coordinate system *L−T* is calculated according to Equation (3), as shown in terms of the following equations:

(12) $${\left[\tilde{E}\right]}_{0^{\circ }}=\left[\begin{array}{ccc} 137.7 & 2.53 & 0\\ 2.53 & 9.04 & 0\\ 0 & 0 & 1.89 \end{array}\right]\times 10^{3}MPa$$

(13) $${\left[\tilde{E}\right]}_{45^{\circ }}=\left[\begin{array}{ccc} 3.98 & 3.6 & -3.42\\ 3.6 & 3.98 & -3.22\\ -3.22 & -3.22 & 3.54 \end{array}\right]\times 10^{4}MPa$$

(14) $${\left[\tilde{E}\right]}_{90^{\circ }}=\left[\begin{array}{ccc} 9.04 & 2.53 & 0\\ 2.53 & 137.7 & 0\\ 0 & 0 & 1.89 \end{array}\right]\times 10^{3}MPa$$

(15) $${\left[\tilde{E}\right]}_{135^{\circ }}=\left[\begin{array}{ccc} 3.98 & 3.6 & 3.42\\ 3.6 & 3.98 & 3.22\\ 3.22 & 3.22 & 3.54 \end{array}\right]\times 10^{4}MPa$$

Herein, the physical properties of *E_L_*, *E_T_*, *G_LT_*, and *v* are calculated as follows: *E_L_* = 40118 MPa, *E_T_* = 40118 MPa, *G_LT_* = 18645 MPa, and *v* = 0.4. Therefore, the dimensionless equivalent stiffness coefficient matrix and the dimensionless influence coefficients matrix of orthotropic materials can be calculated using the following equations:

(16) $$\left[\bar{E}\right]=\left[\begin{array}{ccc} 1.19 & 0.47 & 0\\ 0.47 & 1.19 & 0\\ 0 & 0 & 0.46 \end{array}\right]$$

(17) $$\left[C\right]=\left[\begin{array}{ccc} -0.2983 & 0.1777 & 0\\ 1.8763 & -1.4013 & -9.0933\\ 0.1777 & -0.2983 & 0 \end{array}\right]$$

The results of the residual strain test are presented in Table 5. By substituting these residual strains into Equation (2), the residual stresses (*σ_x_*, *σ_y_*, *τ_xy_*) can be calculated.

**Figure 10:**
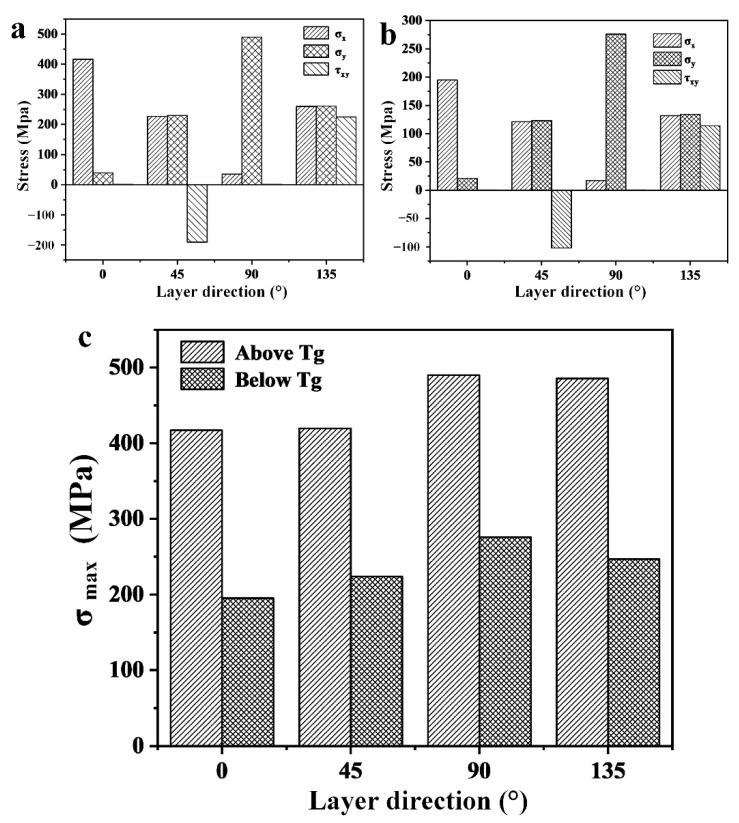
Results of the through-hole drilling method: (**a**) residual stress of specimen drilled at 40 °C; (**b**) residual stress of specimen drilled at −30 °C; and (**c**) maximum primary stress of specimen.

Hereupon, the maximum primary stress can be obtained as shown in Figure 10, according to the following equations:

(18) $$\sigma_{\max}=(\sigma_{x}+\sigma_{y})/2+\sqrt{{\left((\sigma_{x}-\sigma_{y})/2\right)}^{2}+\sigma_{xy}^{2}}$$

(19) $$\phi =\frac{1}{2}\arctan(\frac{2\sigma_{xy}}{\sigma_{x}-\sigma_{y}})$$

Figure 10c demonstrates that the value of maximum residual stress is positive, which proves that the residual stress near the hole is residual tensile stress. Furthermore, the residual stress decreases with the decrease in ET. Therefore, the hole-making process of CFRP (below Tg) is beneficial for the reduction of residual stress.

**Figure 11:**
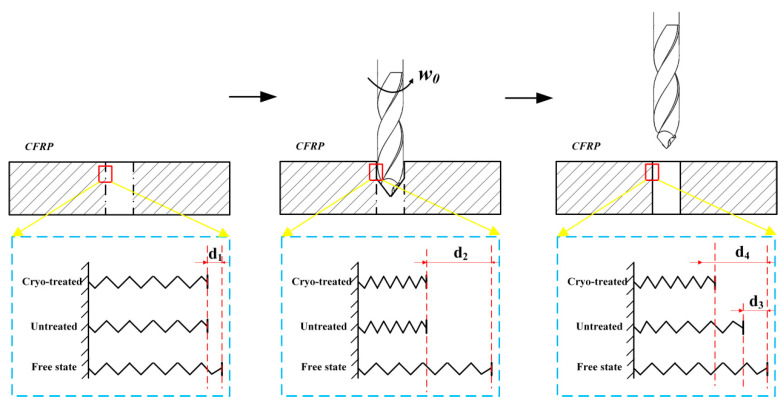
Influence mechanisms of viscoelasticity based on Tg.

Although the machinability of CFRP can be improved through cryogenic treatment, the compressive/compressive deformation resistance property of cryo-treated specimen decreases. Our analysis indicates that the matrix was compressed in the hole-making process when drilled at low temperatures (below Tg), which could not be restored to its initial state due to the viscoelasticity of the matrix (Figure 11), where the relationship of the compressive distance is d2 > d4 > d3 > d1 [40]. Thus, residual stress still remains inside the specimen, which can be proven through the results of the through-hole drilling test.

## 4. Conclusions

The machinability of CFRPs was experimentally investigated by using a cryogenic machining approach with different cutting parameters. Furthermore, the effects of the cryogenic machining method, with DAT lower than Tg and implemented in this study on tensile/tensile and compressive/compressive deformation resistance properties, were investigated, and the following conclusions were obtained from this study:

- Machinability of CFRPs improved when DAT was lower than Tg. The occurrence of damages, such as burr, heat accumulation, etc., decreased due to the cryogenic machining approach.
- Although higher tensile/tensile deformation resistance property was obtained in the cryogenic machining, low compressive/compressive deformation resistance property was obtained simultaneously.
- After ten thousand compressive/compressive fatigue loadings, the residual intensity of specimens under cryogenic treatment was 7.14% lower than those of untreated specimens.
- The residual tensile stress of specimen subjected to cryogenic treatment was lower than those of untreated specimens when using the through-hole drilling method.

First, the finding of this study indicates an improvement in machinability for CFRPs drilled below Tg using the cryogenic treatment method. However, the slight changes in CFRP properties that occur during processing as a result of cryogenic treatment are also extremely important for later service of the component. These results demonstrate the great potential of this study in advanced industrial applications and pave the way for secondary machining operation of CFRP components.

## Figures and Tables

**Table 1:** Dimensions of specimens.

Parameters	Specimen 1	Specimen 2	Specimen 3
Length (mm)	60.4	60.3	59.62
Width (mm)	10.9	11.42	11.38
Thickness (mm)	3.34	3.3	3.28

**Table 2:** Roundness of untreated and cryo-treated CFRP specimens.

Parameters	Above Tg (40 °C)	Below Tg (−30 °C)
Roundness (tensile/tensile fatigue test)	0.02525	0.01068
Roundness (compressive/compressive fatigue test)	0.01324	0.0233

**Table 3:** Residual intensity of untreated and cryo-treated specimens.

Parameters	Above Tg	Below Tg
Residual intensity (tensile/tensile fatigue test) (MPa)	485.91	486.05
Residual intensity (compressive/compressive fatigue test) (MPa)	161.65	150.1

**Table 4:** Mechanical properties of the CFRP plate [39].

Elastic Property	Value	Damage Properties	Value
$E_{11}$(MPa)	137,000	$X_{T}$(MPa)	2000
$E_{22}$(MPa)	9000	$X_{C}$(MPa)	1150
$E_{33}$(MPa)	9000	$Y_{T}$(MPa)	60
$\upsilon_{12}$	0.28	$Y_{C}$(MPa)	152
$\upsilon_{13}$	0.28	$S_{L}$(MPa)	75
$\upsilon_{23}$	0.4	$S_{T}$(MPa)	76
$G_{12}$(MPa)	3780	–	–
$G_{13}$(MPa)	6000	–	–
$G_{23}$(MPa)	6000	–	–
ρ($t/mm^{3}$)	1.79 × 10^−9^	–	–

**Table 5:** Residual strains tested by the through-hole drilling method.

Temperature	$\varepsilon_{1}$(µm)	$\varepsilon_{2}$(µm)	$\varepsilon_{3}$(µm)
40 °C	−741	−554	−72
−30 °C	−438	−234	−57

## Data Availability

Not applicable.

## References

[1] Geier N., Davim J.P., Szalay T. (2019). Advanced cutting tools and technologies for drilling carbon fibre reinforced polymer (CFRP) composites: A review. Compos. Part A Appl. Sci. Manuf..

[2] Vigneshwaran S., Uthayakumar M., Arumugaprabu V. (2018). Review on Machinability of Fiber Reinforced Polymers: A Drilling Approach. Silicon.

[3] Panchagnula K.K., Palaniyandi K. (2018). Drilling on fiber reinforced polymer/nanopolymer composite laminates: A review. J. Mater. Res. Technol..

[4] Romhány G., Kovács L. (2018). Derivation of Ply Specific Stiffness Parameters of Fiber Reinforced Polymer Laminates via Inverse Solution of Classical Laminate Theory. Period. Polytech. Mech. Eng..

[5] Hegde S., Satish Shenoy B., Chethan K.N. (2019). Review on carbon fiber reinforced polymer (CFRP) and their mechanical performance. Mater. Today Proc..

[6] Davim J.P., Reis P. (2005). Damage and dimensional precision on milling carbon fiber-reinforced plastics using design experiments. J. Mater. Process. Technol..

[7] Yan X., Zhang K., Cheng H., Luo B., Hou G. (2019). Force coefficient prediction for drilling of UD-CFRP based on FEM simulation of orthogonal cutting. Int. J. Adv. Manuf. Technol..

[8] Sur G., Erkan Ö. (2020). Surface quality optimization of CFRP plates drilled with standard and step drill bits using TAGUCHI, TOPSIS and AHP method. Eng. Comput..

[9] Raj D.S., Karunamoorthy L. (2018). A new and comprehensive characterisation of tool wear in CFRP drilling using micro-geometry and topography studies on the cutting edge. J. Manuf. Process..

[10] Xu J., Li C., Chen M., El Mansori M., Ren F. (2019). An investigation of drilling high-strength CFRP composites using specialized drills. Int. J. Adv. Manuf. Technol..

[11] Tsao C.C., Hocheng H. (2005). Computerized tomography and C-Scan for measuring delamination in the drilling of composite materials using various drills. Int. J. Mach. Tools Manuf..

[12] Jia Z.Y., Zhang C., Wang F.J., Fu R., Chen C. (2020). An investigation of the effects of step drill geometry on drilling induced delamination and burr of Ti/CFRP stacks. Compos. Struct..

[13] Karpat Y., DeÄŸer B., Bahtiyar O. (2012). Drilling thick fabric woven CFRP laminates with double point angle drills. J. Mater. Process. Technol..

[14] Slamani M., Chatelain J.-F., Hamedanianpour H. (2015). Comparison of two models for predicting tool wear and cutting force components during high speed trimming of CFRP. Int. J. Mater. Form..

[15] Sala G. (2000). Composite degradation due to fluid absorption. Compos. Part B Eng..

[16] Khashaba U.A. (2012). Drilling of polymer matrix composites: A review. J. Compos. Mater..

[17] Weinert K., Kempmann C. (2004). Cutting Temperatures and Their Effects on the Machining Behaviour in Drilling Reinforced Plastic Composites. Adv. Eng. Mater..

[18] Fu R., Jia Z., Wang F., Jin Y., Sun D., Yang L., Cheng D. (2018). Drill-exit temperature characteristics in drilling of UD and MD CFRP composites based on infrared thermography. Int. J. Mach. Tools Manuf..

[19] Xu J., Li C., Chen M., El Mansori M., Paulo Davim J. (2020). On the analysis of temperatures, surface morphologies and tool wear in drilling CFRP/Ti6Al4V stacks under different cutting sequence strategies. Compos. Struct..

[20] Impero F., Dix M., Squillace A., Prisco U., Palumbo B., Tagliaferri F. (2018). A comparison between wet and cryogenic drilling of CFRP/Ti stacks. Mater. Manuf. Process..

[21] Chatterjee A. (2009). Thermal degradation analysis of thermoset resins. J. Appl. Polym. Sci..

[22] Xu J., Li C., Dang J., El Mansori M., Ren F. (2018). A Study on Drilling High-Strength CFRP Laminates: Frictional Heat and Cutting Temperature. Materials.

[23] Abish J., Samal P., Narenther M.S., Kannan C., Balan A.S.S. (2018). Assessment of drilling-induced damage in CFRP under chilled air environment. Mater. Manuf. Process..

[24] John K.M., Kumaran S.T. (2020). A feasible strategy to produce quality holes using temperature-assisted drilling on CFRP. Int. J. Adv. Manuf. Technol..

[25] Sun S., Brandt M., Dargusch M.S. (2010). Thermally enhanced machining of hard-to-machine materialsâ€”A review. Int. J. Mach. Tools Manuf..

[26] Shokrani A., Dhokia V., MuÃ±oz-Escalona P., Newman S.T. (2013). State-of-the-art cryogenic machining and processing. Int. J. Comput. Integr. Manuf..

[27] Basmaci G., Yoruk A.S., Koklu U., Morkavuk S. (2017). Impact of Cryogenic Condition and Drill Diameter on Drilling Performance of CFRP. Appl. Sci..

[28] Rajkumar G.M., Bhardwaj D., Kannan C., Oyyaravelu R., Balan A.S.S. (2018). Effect of chilled air on delamination, induced vibration, burr formation and surface roughness in CFRP drilling: A comparative study. Mater. Res. Express.

[29] Samuel Raj D., Karunamoorthy L. (2019). Performance of cryogenically treated WC drill using tool wear measurements on the cutting edge and hole surface topography when drilling CFRP. Int. J. Refract. Met. Hard Mater..

[30] Kumar D., Gururaja S., Jawahir I.S. (2020). Machinability and surface integrity of adhesively bonded Ti/CFRP/Ti hybrid composite laminates under dry and cryogenic conditions. J. Manuf. Process..

[31] Wang F., Qian B., Jia Z., Cheng D., Fu R. (2018). Effects of cooling position on tool wear reduction of secondary cutting edge corner of one-shot drill bit in drilling CFRP. Int. J. Adv. Manuf. Technol..

[32] Ferreira Batista M., Basso I., de Assis Toti F., Roger Rodrigues A., Ricardo Tarpani J. (2020). Cryogenic drilling of carbon fibre reinforced thermoplastic and thermoset polymers. Compos. Struct..

[33] Zhang L., Tian X., Malakooti M.H., Sodano H.A. (2018). Novel self-healing CFRP composites with high glass transition temperatures. Compos. Sci. Technol..

[34] Melo J.D.D., Radford D.W. (2005). Time and temperature dependence of the viscoelastic properties of CFRP by dynamic mechanical analysis. Compos. Struct..

[35] Morkavuk S., Köklü U., Bağcı M., Gemi L. (2018). Cryogenic machining of carbon fiber reinforced plastic (CFRP) composites and the effects of cryogenic treatment on tensile properties: A comparative study. Compos. Part B Eng..

[36] Wang B., Zhao H., Zhang F., Wang M., Zheng Y. (2021). Comparison of the geometric accuracy of holes made in CFRP/Ti laminate by drilling and helical milling. Int. J. Adv. Manuf. Technol..

[37] Giasin K., Ayvar-Soberanis S. (2017). An Investigation of burrs, chip formation, hole size, circularity and delamination during drilling operation of GLARE using ANOVA. Compos. Struct..

[38] Pagliaro P., Zuccarello B. (2007). Residual Stress Analysis of Orthotropic Materials by the Through-hole Drilling Method. Exp. Mech..

[39] Wang H., Duan Y., Abulizi D., Zhang X. (2017). Design optimization of CFRP stacking sequence using a multi-island genetic algorithms under low-velocity impact loads. J. Wuhan Univ. Technol. Mater. Sci. Ed..

[40] Ahci E., Talreja R. (2006). Characterization of viscoelasticity and damage in high temperature polymer matrix composites. Compos. Sci. Technol..

